# Mechanisms of Maintenance of Foot-and-Mouth Disease Virus Persistence Inferred From Genes Differentially Expressed in Nasopharyngeal Epithelia of Virus Carriers and Non-carriers

**DOI:** 10.3389/fvets.2020.00340

**Published:** 2020-06-19

**Authors:** James J. Zhu, Carolina Stenfeldt, Elizabeth A. Bishop, Jessica A. Canter, Michael Eschbaumer, Luis L. Rodriguez, Jonathan Arzt

**Affiliations:** ^1^USDA-ARS, Foreign Animal Disease Research Unit, Plum Island Animal Disease Center, Orient, NY, United States; ^2^Department of Diagnostic Medicine/Pathobiology, Kansas State University, Manhattan, KS, United States; ^3^Plum Island Animal Disease Center, Oak Ridge Institute for Science and Education (ORISE), Orient, NY, United States; ^4^Institute of Diagnostic Virology, Friedrich-Loeffler-Institut, Greifswald, Germany

**Keywords:** foot-and-mouth disease virus, FMDV, microarray analysis, persistent infection, pharyngeal epithelia, chemokine expression, NFκB signaling pathways, the Th17 response

## Abstract

Foot-and-mouth disease virus (FMDV) causes persistent infection of nasopharyngeal epithelial cells in ~50% of infected ruminants. The mechanisms involved are not clear. This study provides a continued investigation of differentially expressed genes (DEG) identified in a previously published transcriptomic study analyzing micro-dissected epithelial samples from FMDV carriers and non-carriers. Pathway analysis of DEG indicated that immune cell trafficking, cell death and hematological system could be affected by the differential gene expression. Further examination of the DEG identified five downregulated (chemerin, CCL23, CXCL15, CXCL16, and CXCL17) and one upregulated (CCL2) chemokines in carriers compared to non-carriers. The differential expression could reduce the recruitment of neutrophils, antigen-experienced T cells and dendritic cells and increase the migration of macrophages and NK cells to the epithelia in carriers, which was supported by DEG expressed in these immune cells. Downregulated chemokine expression could be mainly due to the inhibition of canonical NFκB signaling based on DEG in the signaling pathways and transcription factor binding sites predicted from the proximal promoters. Additionally, upregulated CD69, IL33, and NID1 and downregulated CASP3, IL17RA, NCR3LG1, TP53BP1, TRAF3, and TRAF6 in carriers could inhibit the Th17 response, NK cell cytotoxicity and apoptosis. Based on our findings, we hypothesize that (1) under-expression of chemokines that recruit neutrophils, antigen-experienced T cells and dendritic cells, (2) blocking NK cell binding to target cells and (3) suppression of apoptosis induced by death receptor signaling, viral RNA, and cell-mediated cytotoxicity in the epithelia compromised virus clearance and allowed FMDV to persist. These hypothesized mechanisms provide novel information for further investigation of persistent FMDV infection.

## Introduction

Foot-and-mouth disease (FMD) is one of the most contagious and economically devastating animal viral diseases. FMD virus (FMDV), a positive-sense single-stranded RNA virus of the family *Picornaviridae* (genus *Aphthovirus*), is the etiological agent of the disease. Susceptible hosts include domesticated and wild cloven-hoofed animals. Infection in cattle initiates via the respiratory tract. During primary infection, the virus replicates locally in the nasopharynx or lungs depending on exposure conditions (1–3). The infection subsequently spreads via the bloodstream (viremia) to secondary replication sites causing typical vesicles at specific regions of the oral cavity, feet, and occasionally other sites. Mortality is generally low in adults, but persistent infection can occur for long periods (30 days−5 years) with virus persisting at the primary infection sites (e.g., nasopharynx) in a high percentage (~50%) of infected cattle, buffalo and sheep (4–8). Specifically, FMDV persistent replication sites in cattle were localized to the epithelial cells of the dorsal soft palate and pharynx (9) and, more precisely, the follicle-associated epithelia of the nasopharyngeal mucosa (7, 10). Although various studies have failed to demonstrate natural transmission from FMDV carrier cattle (8), it has been demonstrated that oropharyngeal fluid from carrier cattle is infectious to naïve cattle (11).

Extensive *in-vivo* studies have been conducted in order to elucidate the mechanisms of persistent FMDV infection in cattle. Zhang and Alexandersen (12) and Zhang et al. (13) showed that declining rate of FMDV RNA levels in oropharyngeal fluid samples during early infection differed between carriers and non-carriers and proposed that differences in the host's abilities to either clear the virus or to support virus replication may determine the establishment of FMDV persistent infection. There was significantly higher anti-FMDV IgA production in carriers than in non-carriers (7, 14, 15), indicating antibodies are not effective in complete clearance of FMDV infection. In addition, the lymphocyte proliferative response of peripheral blood mononuclear cells to FMDV antigens was higher in non-carriers than in carriers (16).

Expression levels of a small number of candidate genes such as cytokines (7, 10, 17, 18) and microRNA (19) have been quantitated in FMDV carriers and non-carriers by qRT-PCR. However, these results do not provide detailed mechanisms involved in persistent infection. Broader transcriptomic studies using microarrays have been conducted to obtain genome-wide expression profiling of tissues targeted for persistent FMDV infection. A transcriptomic analysis showed that the lungs, susceptible to early infection but not persistent infection, expressed significantly higher levels of TNF cytokines and the associated receptors than the pharyngeal tissues that are susceptible to both primary and persistent FMDV infection (20). However, it is unknown if these same differences between the tissues exist between FMDV carriers and non-carriers. Another transcriptomic study of pharyngeal tissues from carriers and non-carriers indicated that inducible regulatory T cells (Treg) especially type 1 regulatory T cells (Tr1) could play a role in persistent infection based on cytokine and Tr1-expressed genes being differentially expressed between carriers and non-carriers (21).

Further transcriptomic investigation using RNA prepared from micro-dissected nasopharyngeal epithelia suggested that persistent FMDV infection is associated with compromised apoptosis and a reduced cellular immune response based on some most-differently expressed genes (22). These results could further explain the differences between carriers and non-carriers. Immunohistochemistry analysis using anti-CD3, anti-CD8, and anti-γδTCR antibodies showed no differences in the numbers of detected cell populations between carriers and non-carriers (22). The current study is a continued analysis of all differentially expressed genes (DEG) from previously published expression data (22) derived from micro-dissected nasopharyngeal epithelium samples of FMDV carriers and non-carriers during the persistent phase of FMDV infection in order to identify additional mechanisms involved. Pathway analyses using the list of all detected DEG show that genes involved in immune cell trafficking were over-represented by DEG including four chemokines known to play key roles in mucosal immunity. Other immune-related DEG support the downregulated chemokine expression in carriers and suggest that reduced recruitment of neutrophils, antigen-experienced T cells and dendritic cells in carriers could lead to compromised virus clearance and allow FMDV to persist.

## Methods and Materials

### Gene Expression Data

The microarray data used in this study and the details of the animal experiments have been reported (21, 22). The data were produced using a custom bovine gene expression 60-mer oligonucleotide microarray designed based on gene expression information displayed on the bovine genome in the UCSC Genome Browser (https://genome.ucsc.edu/index.html). Microarrays and reagents were manufactured by Agilent Technologies (San Jose, CA) and the lab procedures were conducted based on the protocols and equipment recommended by the manufacturer. For comparison of the gene expression levels between carriers and non-carriers, microarray expression data from the micro-dissected pharyngeal epithelia of three carriers (persistently infected by FMDV A24 for >28 days) were compared to those from the corresponding micro-dissected tissues of four non-carriers that had cleared FMDV as reported by Stenfeldt et al. (22). For comparison of gene expression between the micro-dissected pharyngeal epithelia samples and whole tissue macerates of nasopharyngeal samples from the corresponding anatomical site, normalized mean expression data from the micro-dissected pharyngeal epithelia of sixteen animals (22) were compared to the data from the whole tissue macerates of nineteen cattle as reported by Eschbaumer et al. (21).

### Statistical Analysis

R scripts implemented with the LIMMA package (23) were used to normalize and analyze the microarray data as previously described (21). All signal intensities (averaged photons per pixel) used in the statistical analysis were Log_2_ transformed. Genes differentially expressed between carriers and non-carriers with a false discovery rate (FDR) of 0.10 or smaller and an expression difference of at least 50% were considered as statistically significant genes in the transcriptomic study. This FDR significance threshold increases the detection power (fewer false negatives/type II errors) with a false positive (type I error) rate of 0.10 in declared DEG, or one false positive in 10 DEG, compared to FDR at 0.05 (one in twenty) to balance type I and type II errors. The means of normalized signal intensities (photons per pixel) of ACTA1, ACTA2, and ACTB were used as the internal controls to normalize the expression data to account for differences in the methods of data acquisition between micro-dissected epithelia (22) and whole tissue macerates (21).

### Pathway Analysis

All bovine genes included in the microarray design were mapped to human reference genes using computer analysis via NCBI BLAST and/or manual annotation by aligning the microarray probe sequences on bovine genome sequences displayed on the UCSC Genome Browser using BLAT program (https://genome.ucsc.edu/cgi-bin/hgGateway). The list of upregulated and downregulated genes associated with the human Entrez Gene ID was analyzed with Ingenuity Pathway Analysis (IPA) (Qiagen, Maryland) and a NCBI Functional Annotation Bioinformatics Microarray Analysis program (DAVID Bioinformatics Resources version 6.8) to identify the biological pathways significantly over-represented by DEG. The biological functions of DEG were based on scientific publications obtained from the PubMed website (https://www.ncbi.nlm.nih.gov/pubmed/) listed as cited references or the NCBI Gene database (https://www.ncbi.nlm.nih.gov/gene/) listed as NCBI.

### Biological Inferences

Biological inferences were based on (i) reported biological functions of DEG, (ii) gene expression levels based on microarray averaged signal intensity and (iii) magnitudes (fold difference) of upregulated or downregulated expression, assuming that (1) genes with a higher signal intensity and larger differential expression play a bigger biological role in their gene group and (2) upregulated expression enhances gene activities and vice versa. Differential expression of genes with cell-specific expression was also used to infer the differences in the number of the cells. Genes with no significant differential expression (FDR > 0.10) but known to play important roles in the relevant biological pathways/processes associated with DEG were also used as references or supporting results for DEG and/or DEG related mechanisms. Probabilities of differential expression at gene levels are listed as *P*-values along with FDR. Genes downregulated or upregulated in carriers compared to non-carriers were expressed as negative and positive values (fold changes), respectively. Multiple DEG involved in a known immune mechanism were used in the formulation of hypothesis. All immune mechanisms known to play roles in virus clearance based on our literature review were considered for candidate mechanisms.

### Proximal Promoter Analysis

The nucleotide sequences up- and down-stream of the gene transcription start sites of four mucosal chemokines (RARRES2/chemerin, CXCL15, CXCL16, and CXCL17) were downloaded from the UCSC Genome Browser (http://genome.ucsc.edu/cgi-bin/). The core promoters were predicted using Neural Network Promoter Prediction (NNPP version 2.2 software) (24). The proximal promoters, 500-bp up-stream and 250-bp down-stream nucleotide sequences of the TATA box or CpG island sequences associated with the transcription starting sites, were used in the prediction of transcription factor binding sites (TFBS) using the TESS 2.0 program (25) and JASPAR database (http://jaspar.genereg.net/downloads/) with a log-odd score of 7.0 or higher (default value at 6.0). Over-lapping TFBS for the same transcription factors were counted if the sites differed by at least five nucleotides. AP-1, CREBs, NFκB, IRF3, and STAT1/STAT2 were selected to represent the transcription factors activated by MAPK, NFκB, IRF3 and interferon signaling pathways.

## Results

### Pathway Analysis

**Figure 1:**
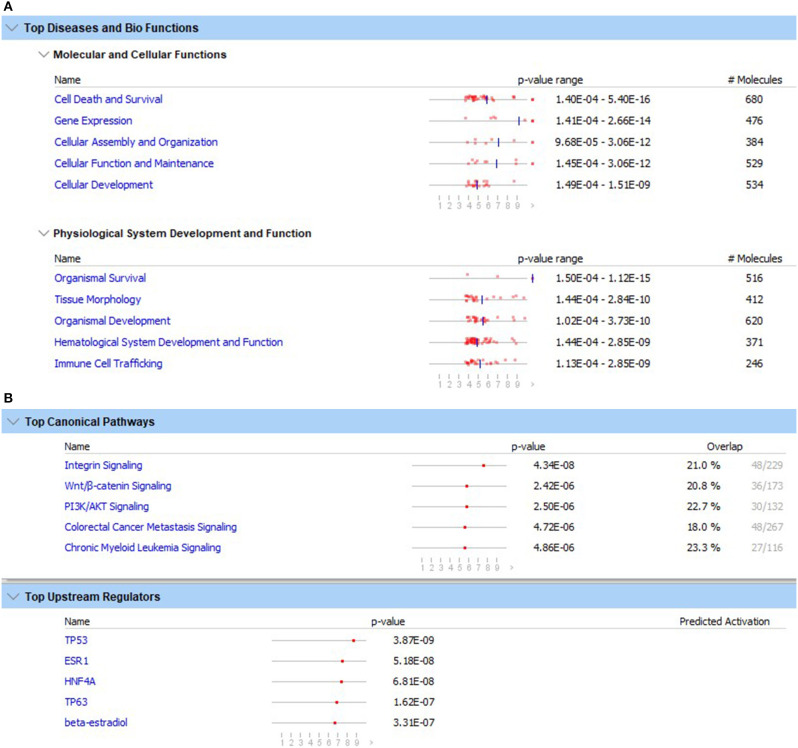
Top five diseases and biological functions **(A)** and top five canonical pathways and upstream regulators **(B)** with the lowest likelihoods (*p*-value) of the associations/overlaps between the differentially expressed gene set (both up- and down-regulated) and the pathways/biological processes by random chances in the Qiagen Ingenuity Pathway Analysis using the list containing ENTREZ numbers and up- and down-regulated DEG. The dots in horizontal lines are the negative log transformation of *p*-values.

There were 1,505 probes with significantly downregulated expression in carriers compared to non-carriers and 1,097 probes with upregulated expression. Among the genes associated with these probes, there were 1,281 downregulated and 951 upregulated genes that could be mapped to human or mouse genes. This gene set including both up- and down-regulated genes was used in the NCBI DAVID and IPA pathway analyses. The KEGG and REACTOME pathway analyses using the NCBI DAVID program showed no significant pathways associated with these DEG. In contrast, pathway analysis with the IPA program detected several significant associations. The top five canonical pathways significantly over-represented by DEG were (1) integrin signaling, (2) Wnt/β-catenin signaling, (3) PI3K/AKT signaling, (4) colorectal cancer metastasis signaling, and (5) chronic myeloid leukemia signaling (Figure 1A). The top five upstream regulators were TP53, ESR1, HNF4A, TP63, and beta-estradiol (ordered based on probability) (Figure 1A). None of the top upstream regulator genes were differentially expressed between carriers and non-carriers.

The top five molecular and cellular functions that were significantly associated with the differential expression were (1) cell death and survival, (2) gene expression, (3) cellular assembly and organization, (4) cellular function and maintenance, and (5) cellular development (Figure 1B). The top five inferred physiological system development and function were (1) organism survival, (2) tissue morphology, (3) organism development, (4) hematological system development and function, and (5) immune cell trafficking (Figure 1B). Because cell death/apoptosis and immune cell development, function and trafficking play important roles in immunity against virus infections, DEG involved in these functions were examined in detail.

Among 24 functions associated with immune cell trafficking (Table 1), the top five functions with the lowest *p*-values ranging from 3.05E-07 to 2.85E-09 were associated with leukocyte movement/infiltration and neutrophil movement. Based on these findings, the differential expression of chemokines was further examined as listed in Table 2, followed by differential expression of the genes supporting or regulating the differential chemokine expression in Table 3. For DEG involved in immune cell development and function, differential expression of cytokines, cytokine signaling pathways and other immune regulatory genes together with their function was analyzed (Tables 4–6). The DEG involved in apoptosis were evaluated for their potential roles in affecting apoptosis induced by viral RNA, death receptor signaling and cell-mediated cytotoxicity as in Table 7. These DEG were used to formulate the candidate immune mechanisms involved in the maintenance of FMDV persistent infection as shown in **Figure 3**. The TFBS in proximal promoter regions (Table 8) were predicted as the supporting results of the hypothesis.

**Table 1:** The *p*-values of the number (#) of differentially expressed genes (DEG) in 24 functions (top five with lowest *p*-value in bold fonts) of immune cell trafficking by random chances in the analyses of diseases and functions using Qiagen Ingenuity Pathway Analysis program.

**Function**	**Diseases or functions annotation**	***p*-value**	**# of DEG**
Accumulation	Accumulation of neutrophils	1.13E-04	20
Activation	Activation of leukocytes	1.33E-05	129
	Activation of lymphocytes	1.67E-05	89
	Activation of mononuclear leukocytes	2.37E-05	92
Adhesion	Adhesion of immune cells	5.11E-05	70
	Adhesion of mononuclear leukocytes	5.85E-05	33
Cell movement	Cell movement of leukocytes	**2.85E-09**	169
	Cell movement of phagocytes	1.60E-07	121
	Cell movement of neutrophils	**3.05E-07**	71
	Cell movement of granulocytes	2.03E-06	81
	Cell movement of mononuclear leukocytes	5.64E-05	93
	Cell movement of antigen presenting cells	9.20E-05	73
Cell rolling	Cell rolling of leukocytes	3.90E-05	20
	Cell rolling of phagocytes	1.09E-04	11
Cellular infiltration	Cellular infiltration by leukocytes	**7.11E-07**	89
	Cellular infiltration by phagocytes	4.18E-06	63
	Infiltration by neutrophils	7.32E-05	39
	Cellular infiltration by macrophages	1.08E-04	39
Chemotaxis	Chemotaxis of leukocytes	1.87E-05	73
	Chemotaxis of phagocytes	6.74E-05	60
	Chemotaxis of neutrophils	1.11E-04	35
Homing	Homing of leukocytes	**6.63E-07**	82
	Homing of mononuclear leukocytes	8.38E-05	44
Migration	Leukocyte migration	**1.23E-08**	196

**Table 2:** Mean expression levels (microarray signal intensity, MSI), false discovery rates (FDR), fold differences (+ and – values as up- and down-regulated in carriers compared to non-carriers, respectively) and chemotactic activities of chemokine genes differentially expressed between the nasopharynx epithelia of carriers and non-carriers.

**Gene**	**MSI**	**FDR**	**Fold**	**Chemotactic activity^a^**	**References**
CCL2	841	0.03	6.8	Inflammatory monocytes and NK cells	(26–28)
CCL23	422	0.10	−17.9	Resting T cells, monocytes & neutrophils	(29)
Chemerin	1188	0.02	−5.7	Immature DC and macrophages (Mϕ)	(30, 31)
CXCL15	525	0.08	−7.6	Neutrophils	(32)
CXCL16	13128	0.04	−2.5	Activated CD8+/CD4+ T, IEL and NKT cells	(33–35)
CXCL17	3028	0.01	−3.5	Immature DC, Mϕ, CD8+ Tem and Trm cells	(36)
ELR+CXCLs^b^	2530	n/a	−2.4	Neutrophil > monocyte, NK, CD8+ T cells	(27)

a *DC, dendritic cells; Mϕ, macrophages; IEL, intraepithelial lymphocytes; NKT, natural killer T cells; Tem, memory T effector; Trm, resident memory T cells*.

b *ELR+CXCLs: CXCL1, CXCL2, CXCL3, CXCL5, CXCL8, and CXCL15, which total signal intensity is the sum of the signal intensity of each ELR+CXCL chemokine in carriers and non-carriers*.

**Table 3:** Mean expression levels (microarray signal intensity, MSI), false discovery rates (FDR) and fold differences (+ and – values as up- and down-regulated in carriers compared to non-carriers, respectively) of differentially expressed genes supporting decreased numbers of neutrophils, antigen-experienced T cells and dendritic cells and increased number of naïve and natural intraepithelial lymphocytes.

**Group**	**Gene**	**MSI**	**FDR**	**Fold**	**Expressing cells/functions^a^**	**References**
Neutrophil	C3	16129	0.03	−15	Neutrophil chemoattractant C3a	(37)
	CD38	241	0.02	−8.1	Neutrophils/migration	(30, 38, 39)
	CTSC	23238	0.04	−2.6	Neutrophils/granule component	NCBI
	CTSF	1084	0.01	−11.7		
	CTSV	5645	0.04	−3.4		
	CXCR1	102	0.09	−2.3	Neutrophils/ELR+ CXCL receptor	(27, 40)
	CXCR2	259	0.06	−3.8		
	RGS5	426	0.06	5.4	Neutrophils/migration inhibitor	(41)
Antigen-specificT cell	CD27	556	0.00	−4.4	CD8+ effector T cells	(42)
	CD44	13684	0.07	−2.9	Antigen-experienced T cells	(43)
	CD73	1244	0.05	−17.0	CD8+ effector T cells and Treg	(44–46)
	CD244/2B4	450	0.03	−4.9	NK cells and CD8+ effector T cells	(47, 48)
	LAT	248	0.02	−6.9	T cells/TCR signaling	(49–51)
	ITK	1469	0.09	−3.5	T cells/TCR signaling	(51)
	TRBC2	1074	0.09	−5.1	T cells/TCR beta chain	(49–51)
	TRBC2	1113	0.05	−3.4		
	CD45R	360	0.01	14.3	Naïve T cells	(52, 53)
	SELL/CD62L	1747	0.01	4.3	Naïve T and central Tm cells	(52, 53)
	CD2	716	0.31	−5.3	T cells	NCBI
	CD3D	1700	0.59	2.0	T cells	NCBI
	CD4	420	0.72	−1.4	CD4+ T cells	NCBI
	CD8A	1601	0.82	−1.5	CD8+ T and IEL	NCBI
	CD8B	120	0.65	1.8	CD8+ T cells	NCBI
Antigen-non-specific T cells	CD16	89	0.02	2.1	NK cells and monocytes	(54)
	GZMA	91	0.05	4.6	NK cells, cytotoxic T cells, Treg	NCBI
	GZMM	66	0.08	2.5		
	KLRB1/CD161	111	0.09	4.0	NK cells and natural IEL	(55)
	KLR (unknown)	164	0.00	3.8		
	NCR2	625	0.00	10.4	NK cell receptors	(56)
	NCR3	243	0.27	4.4		
Dendritic cells	CD276/B7-H3	228	0.04	−2.7	Antigen presenting cells	(57)
	CLEC9A/DNGR1	93	0.07	−2.2	DC/cross-presentation	(58)
	VISTA/B7-H5	1182	0.05	−5.0	Antigen presenting cells	(59)
	VTCN1/B7-H4	2468	0.04	−5.0	Antigen presenting cells	(60)
Macro-phages	C5AR1	62	0.01	2.0	Myeloid cells, Mϕ M1 activation	(37)
	CD300A	66	0.02	1.6	Macrophages	(61)
	CD300LD	154	0.06	2.5	Macrophages	(61)
	MMD	914	0.03	9.3	Macrophages	(62)

a *DC, dendritic cells; IELs, intraepithelial lymphocytes; Mϕ, macrophage; TCR, T cell receptor*.

**Table 4:** Mean expression levels (microarray signal intensity, MSI), false discovery rates (FDR) and fold differences (+ and –values as up- and down-regulated in carriers compared to non-carriers, respectively) of cytokines and the receptors differentially expressed between the nasopharynx epithelia of carriers and non-carriers.

**Gene group**	**Gene**	**MSI**	***p*-value**	**FDR**	**FD**	**Function**
IFN, Type 1	IFNA	70		0.10	1.5	Anti-viral cytokines
IL-1 family	IL1A	102		0.04	6.0	Proinflammatory
	IL36A	104		0.00	9.9	
	IL1RN	5405	0.02	0.18	1.8	Anti-inflammatory or stimulate Treg cells
	IL33/IL1F11	578		0.02	14.7	
	IL1RL1/ST2	55	0.02	0.18	2.2	
	sIL1RAP	115		0.07	4.2	
IL-2 family	IL7	139		0.04	8.2	T & B cell development
	IL7R	3099	0.07	0.33	2.1	IL7 receptor
	IL15	100		0.06	1.7	↑NK & CD8+ T cells
	IL15R	146	0.01	0.12	3.3	IL-15 receptor
IL-10 family	IFNL	184		0.04	2.5	Anti-viral cytokines
	IL10	112	0.92	0.98	−1.1	Immune inhibition
	IL10RA	3866		0.06	2.9	
	IL22	42	0.04	0.25	1.6	↑antimicrobial proteins
TNF family	TNF	494		0.00	15.0	Inflammation/apoptosis
	TNFSF10/TRAIL	305		0.04	2.3	Apoptosis
	TNFSF15/TL1A	401		0.10	7.1	Inflammation/apoptosis
Th17-related	IL6	139	0.02	0.19	1.3	↑Th17 differentiation
	IL6R	2696	0.01	0.13	4.5	IL-6 receptor
	IL23A	540	0.01	0.11	4.2	↑Th17 differentiation
	IL12RB1	387	0.02	0.19	−7.0	IL-23 receptor
	Il17A	161	0.12	0.34	3.4	↑Th17 response
	IL17F	227	0.15	0.49	−1.9	
	IL17RA	951		0.06	−2.4	IL17A and IL17F receptor
Anti-inflammatory	EBI3/IL35B	83	0.02	0.21	3.6	Immune suppression

**Table 5:** Mean expression levels (microarray signal intensity, MSI), false discovery rates (FDR) and fold differences (+ and –values as up- and down-regulated in carriers compared to non-carriers, respectively) of differentially expressed genes in interferon, IRF3, MAP, and NFκB signaling pathways.

**Gene group**	**Gene**	**MSI**	**FDR**	**Fold**	**Function**	**References**
TLR	TLR6	147	0.05	−7.7	Activate NFκB, IRFs and MAPKs	NCBI
Signal transducers	AKT1	2378	0.05	−2.5	Enhance interferon signaling & NFκB activation	(63, 64)
	AKT3	1000	0.01	−6.3		
	CHUK/IKKα	326	0.06	3.1	Non-canonical NFκB signaling	(65)
	IKBKB/IKKβ	6116	0.12	−4.3	Canonical NFκB signaling	
	IKBKG/IKKγ	1693	0.15	−3.3	Canonical NFκB signaling	
	MAP3K9	767	0.01	−5	MAP kinases in MAP signaling pathways	NCBI (66)
	MAPK3	1810	0.06	−10.6		
	MAPK8IP1	168	0.03	−1.8		
	MAPKAPK3	20022	0.09	−1.8		
	MAPKAPK5	537	0.08	−5.1		
	TAB1	1366	0.03	−2.9	Signaling transducers in IRF3, MAP and NFκB signaling pathways	(64–67)
	TRAF3	1980	0.02	−3.4		
	TRAF6	267	0.00	−12.4		
NFκB signaling enhancers	OTUB1	2939	0.01	−4.9	Specific ubiquitin iso-peptidase	(68)
	RNF128	1467	0.00	−4.3	E3 ubiquitin ligase	(69)
	TGFB2-OT1	226	0.05	−3.6	Activate NFκB RELA	(70)
	TRIM52	128	0.03	−3.1	Enhance NFκB signaling	(71)
NFκB signaling inhibitors	HIVEP2	1033	0.05	11.8	Inhibit NFκB in DNA binding	(72)
	NKIRAS1	318	0.03	4.6	Inhibit IKKβ activity	(73)
	NLK	375	0.03	9.7	Inhibit co-activators of NFκB	(74)
	NKRF	147	0.07	2.8	Nuclear inhibitor of NFκB	(75)
Transcription factors	CREB5	264	0.00	−22.5	Co-activated with NFκB by TLR	(65, 66)
	NFKB2	2376	0.03	−4.1	Non-canonical NFκB signaling	(65)
	RELA	4183	0.08	3.8	Canonical NFκB signaling	(65)
miRNAs	MIR155HG	219	0.00	12.7	Suppress interferon signaling	(76)
	MIR221	540	0.05	2.9	↓ IFNβ expression & LPS signaling	(77, 78)
	MIR503HG	165	0.05	2.4	Inhibit NFκB signaling	(79)

**Table 6:** Mean expression levels (microarray signal intensity, MSI), false discovery rates (FDR) and fold differences (+ and –values as up- and down-regulated in carriers compared to non-carriers, respectively) of genes regulating or expressed on immune cells.

**Group**	**Gene**	**MSI**	***P***	**FDR**	**Fold**	**Functions**	**References**
Macrophage	MFSD6	2626		0.03	−3.9	MHC-I restricted killing by Mϕ	(80)
	MHCIb	1019		0.09	−7.7	Non-classical MHC Class I	NCBI
NK cell	NCR3LG1	616		0.07	−4.8	Membrane NCR3 ligand	(81)
	NID1	346		0.01	12.1	Extracellular NCR2 ligand	(82)
T cells or intraepithelial lymphocytes (IEL)	CD69	121		0.00	5.7	Inhibit Th17 and CD8+ Teff cells, stimulate Treg cells and increase tryptophan uptake	(82, 83)
	CD69	268		0.00	4.9		
	CD69	277		0.00	4.9		
	CD98	3005	0.20	0.55	−1.6	Transport of tryptophan	(83)
	LAT1	7268		0.05	−2.8		
	CD27	556		0.00	−4.4	CD8+ T cell cytotoxicity	(42)
	CD244/2B4	450		0.03	−4.9	NK and CD8+ T cell cytotoxicity	(47, 48)
	TNFRSF19	1468		0.00	−9.4	↓TGFβ effect on CD8+ cells	(84, 85)
	IGHD	587		0.01	2.3	IgD on γδ T cells	(86)
	CD103	3984	0.03	0.21	−2.5	Resident memory T cells	(87)
	T-bet	564		0.05	4.5	↑ in non-helped Trm	(88, 89)
Th17 cell	RORC	748	0.03	0.24	−5.9	Th17 transcription factor	NCBI
	STAT3	17482	0.01	0.13	−1.5	Promote Th17 differentiation	(90)
	TIAM1	3826		0.00	−20.8	Activate IL-17 promoter	(91)
	ETS1	881		0.01	4.7	Suppress Th17 differentiation	(92)
	IL1RL1/ST2	55	0.02	0.18	2.2	↑ on stimulated Th17	(93)
	IL22	42	0.04	0.25	1.6	Inhibit Th17 response	(94)
	IL33	578		0.02	14.7	↓ Th17	(95)
	STAT5B	2118		0.08	4.4	Inhibit Th17 cell differentiation	(90)
Tr1 cell	CD49B	216		0.05	4.0	Tr1 cell marker	(96)
	EBI3/IL27B	83	0.02	0.21	3.6	Stimulate Tr1 differentiation	(96)
	MAF	1729	0.01	0.13	2.1	Tr1 transcription factor	(96)
	PRDM1	2302		0.08	2.9	Tr1 cell marker	(97)
Treg cell	FOXP3	116		0.07	−2.1	Treg transcription factor	(98)
	ThPOK	3191		0.09	−2.8	CD4+ transcription factor	(98)

**Table 7:** Mean expression levels (microarray signal intensity, MSI), false discovery rates (FDR) and fold differences (+ and – values as up- and down-regulated in carriers compared to non-carriers, respectively) of genes involved in apoptosis or inflammatory mediator production.

**Group**	**Gene**	**MSI**	***P***	**FDR**	**Fold**	**Functions**	**References**
Apoptosis	CASP3	5887		0.07	−6.9	Apoptosis activating caspase	(99)
	BNIP2	708		0.01	9.2	Anti-apoptosis	NCBI
	BNIPL	30865		0.04	−3.2	Pro-apoptosis	NCBI
	BCL2L14	832		0.02	−8.3	Pro-apoptosis	NCBI
	BCL2L1	1371		0.02	−8.2	Pro-apoptosis	NCBI
	TP53BP1	1386		0.06	−7.2	Pro-apoptosis via NFκB	(100)
	TP53RK	1042		0.07	−7.3	Pro-apoptosis via TP53	(101)
Inflammatory mediators	IGHE	66	0.02	0.16	3.9	IgE heavy chain	NCBI
	LTB4R	170	0.02	0.18	5.7	Leukotriene B4 receptor 1	(102)
	PTGR1_s	1554		0.00	−10.3	Inactivation of leukotriene B4	NCBI
	PTGR1_l	259	0.41	0.74	−1.8		
	PLA2G2A	161		0.00	18.5	Production of leukotriene B4 and prostaglandins	(103–105)
	PLA2G2A	211		0.00	15.7		
	PTGES	240	0.01	0.11	−2.6	Prostaglandin E synthesis	
	PTGES2	715		0.00	−7.1		
	PTGS1	1499	0.04	0.27	−3.3	Prostaglandin G/H synthesis	

**Table 8:** The numbers and locations of predicted transcription factor (TF) binding sites (TFBS) in the proximal promoters (CpG island or 500 bp upstream and 250 bp downstream of TATA box) of chemerin, CXCL15, CXCL16, and CXCL17 genes.

**Gene**	**Type**	**TF^a^**	**TFBS**	**TFBS Location in the proximal promoters^b^**
Chemerin	CpG	NFkB1	5	80–92, 229–241, 264–276, 296–308, 328–340
		RELA	2	81–91, 523–532
CXCL15	TATA	IRF3	6	220–240, 277–297, 305–325, 664–684, 690–710, 730–750
		RELA	1	281–290
		STAT1::STAT2	4	304–318, 372–386, 665–679, 731–746
CXCL16	TATA and CpG	CREB5	1	124–135
		FOS::JUN	1	124–135
		IRF3	5	409–429, 414–434, 420–440, 426–446, 698–706
		NFKB1	1	519–531
		RELA	2	519–528, 789–798
		STAT1::STST2	3	419–433, 525–539, 693–707
CXCL17	TATA	FOS::JUN	1	70–82
		IRF3	2	363–383, 381–401
		RELA	1	388–397
		STAT1::STAT2	3	364–378, 388–402, 524–537

a *Underlines indicate that TFBS are present in all proximal promoters*.

b *Underlines indicate overlapping of TFBS for different transcription factors in the proximal promoter*.

### Chemokines and the Receptors

Six chemokine genes were differentially expressed between carriers and non-carriers. Only one of these chemokines (CCL2) was significantly upregulated by 6.8-fold in carriers compared to non-carriers (Table 2). CCL2 has chemotactic activity for inflammatory monocytes and NK cells (26–28). Additionally, higher expression of C5AR1, CD16, CD300A, CD300LD, GZMA, GZMM, KLRB1, MMD, and NCR2 including an unannotated KLR in carriers compared to non-carriers (Table 3) supports increased migration of monocytes and NK cells into the persistently infected epithelia. Monocyte to macrophage differentiation-associated (MMD) is highly expressed in mature differentiated macrophages after migration to tissues but is absent in monocytes (106). CD16, specifically expressed on NK cells and some monocytes/macrophages, can activate antibody directed cell-mediated cytotoxicity (54, 107), whereas KLR receptors and NCR2 are receptors highly expressed on NK cells (56). Granzymes are cytotoxicity effectors expressed in cytotoxic T cells and NK cells (108) and Treg cells (109). C5AR1 is expressed in myeloid cells including macrophages and granulocytes (37). CD300 receptors which are mainly expressed on myeloid cells play a fundamental role in immune regulation (61).

**Figure 2:**
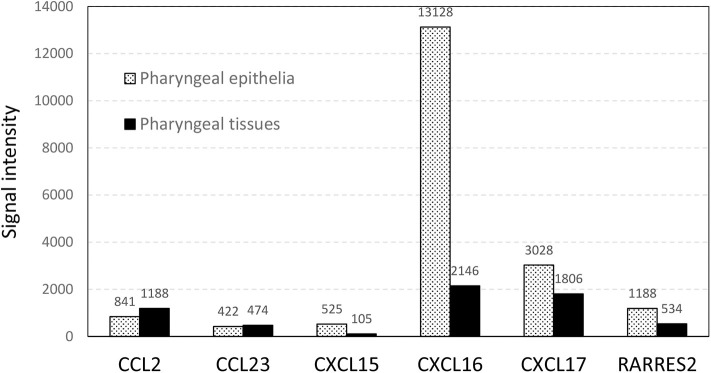
The normalized microarray signal intensity (photons per pixel) of six differentially expressed chemokines in total RNA prepared from micro-dissected pharyngeal epithelia and whole pharyngeal tissues.

The expression of five chemokines (CCL23, CXCL15, CXCL16, CXCL17, and RARRES2, also named chemerin) was significantly downregulated in carriers compared to non-carriers by 2.5- to 17.9-fold (Table 2). These chemokines all have chemotactic activities for antigen presenting cells, antigen-experienced T cells, monocytes, NKT cells and/or neutrophils (29–36). Chemerin, CXCL15, CXCL16, and CXCL17 were expressed at higher levels by 2.2-, 5.0-, 6.1-, and 1.7-fold, respectively, in the micro-dissected epithelia than in the whole tissues (Figure 2), indicating epithelium-specific expression. The differential expression of chemokines could potentially result in reduced recruitment of dendritic cells, antigen-experienced T cells and neutrophils to the nasopharyngeal epithelium of the carriers compared to the non-carriers. This hypothesis was further supported by analysis of DEG associated with expression of the chemokines of interest (Table 3).

Two chemokine receptors, CXCR1 and CXCR2, known to be predominately expressed on neutrophils (27, 40), were expressed at significantly lower levels in carriers than in non-carriers (Table 3). Cathepsins are components of neutrophil granules, and the expression of three cathepsins (CTSC, CTSF, and CTSV) was significantly lower in carriers than in non-carriers. CD38 is important for neutrophil migration (38, 39), whereas RGS5 inhibits neutrophil chemotaxis and trafficking (41). CD38 expression was lower and RGS5 was higher in carriers than in non-carriers. The expression of C3, an important component for activation of the classical and alternative complement activation pathways that lead to production of C5a to recruit neutrophils (110), was downregulated by 15- fold in carriers compared to non-carriers (Table 3). All ELR+ CXCLs have chemotactic activity for neutrophils (27, 32). Although only CXCL15 showed significant differential expression, the total normalized signal intensity of six bovine ELR+ CXCLs including CXCL1, CXCL2, CXCL3, CXCL5, CXCL8 and CXCL15 was 2.4 times lower in carriers than non-carriers (Table 2), further supporting lower expression of neutrophil-recruiting chemokines in carriers.

Other DEG supported reduced recruitment of antigen-experienced T cells and dendritic cells in nasopharyngeal epithelium of FMDV carriers (Table 3). CD44 is a cell marker for antigen-experienced T cells including T effector, central memory T, T effector memory and T resident memory cells but not naïve T cells (43). CD44 expression was downregulated by nearly threefold in carriers compared to non-carriers. There were three effector T-cell-expressed genes (CD27, CD73 and CD244 (42, 44–48) with ≥ 4.4-fold decreased expression in carriers. LAT, IKT and two TRBC2 (T cell receptor beta constant 2) involved in TCR signaling (49–51) displayed >3-fold downregulated expression in carriers. By contrast, genes highly expressed on naïve T and natural intraepithelial lymphocytes such as CD45R, CD62L, and CD161 (52, 53, 55) were expressed significantly higher in carriers than in non-carriers. The expression of T cell marker genes, CD2, CD3D, CD4, CD8A, and CD8B was not significantly different. Dendritic cells express CLEC9A/DNGR1, CD276, VISTA and VTCN1 (57–60). These four genes were expressed at significantly lower levels in carriers than in non-carriers. These results suggest a reduced recruitment of dendritic cells and antigen-experienced T cells within the nasopharyngeal epithelium of FMDV carriers.

### Cytokines and the Receptors

Ten cytokines belonging to interferon (IFNA and IFNL), IL-1 (IL1A, IL33, and IL36A), IL-2- (IL7 and IL15) or TNF (TNF, TNFSF10, and TNFSF15) families were expressed at significantly higher levels in carriers than in non-carriers (Table 4). All significantly upregulated cytokines were proinflammatory or immune-stimulatory except IL33. IL33 activates Th1 and Th2 cells, group 2 innate lymphoid cells, and CD8+ T cells, and it also plays a key role in suppressing Th17 and promoting Treg (95). Among the significant cytokines, TNF and IL33 were expressed at the highest levels and were the most upregulated (approximately 15-fold). Other cytokines significantly upregulated by ~6- to 10-fold were IL1A, IL7, IL36A, and TNFSF15, whereas IFNA, IFNL, IL15, and TNFSF10 were expressed at levels 1.5- to 2.5-fold higher in carriers than in non-carriers (FDR ≤ 0.1). The signaling of IL-1 cytokines can be inhibited by soluble IL1RAP (sIL1RAP, an alternative 3' end transcript) (111), and the sIL1RAP expression level was significantly higher in carriers than in non-carriers (the only differently expressed receptor of the cytokine DEG) was not differentially expressed between carriers and non-carriers. The expression of a receptor (IL10RA) of IL10, an immunosuppressive cytokine (112), was nearly 3-fold higher in carriers than in non-carriers. These results suggest that 15-fold upregulation of IL33 could significantly suppress the Th17 response.

There are also several cytokine genes differentially expressed at gene levels (*P* ≤ 0.05). IL1RN is an IL-1 antagonist. IL6 and IL23 are important cytokines stimulating Th17 cell differentiation (113). IL22 plays a key role in mucosal immunity by stimulating inflammatory responses and inducing S100s and defensin expression (94). EBI3 or IL35B is a part of an immune inhibitory cytokine, IL35. The expression of IL1RN, IL6, IL22, IL23A, and EBI3/IL35B was upregulated in carriers at *p* ≤ 0.05. Among the receptors of the differentially expressed cytokines, IL6R, IL15R, and IL1RL1 (ST2, IL33 receptor) were expressed at higher levels (*p* ≤ 0.02) in carriers than in non-carriers, whereas IL12RB1 (a part of IL23 receptors) was expressed at a lower level (*p* = 0.02; FDR = 0.19) in carriers than in non-carriers. The expression of IL10 was different between carriers and non-carriers.

### Signal Transducing Genes

There were several DEG in interferon, IRF3, MAPK and NFκB signaling pathways that could negatively impact cytokine signaling in carriers (Table 5). Toll-like receptor 6 (TLR6) was expressed 7.7-fold lower in carriers compared to non-carriers. The expression of ATK1 and AKT3, which are involved in interferon signaling (63, 114), were significantly lower in carriers than in non-carriers. There were eight signal transducers (MAP3K9, MAPK3, MAPK8IP1, MAPKAPK3, MAPKAPK5, TAB1, TRAF3, and TRAF6) and two transcription factors (CREB5 and NFKB2) in the IRF3, MAPK and NFκB signaling pathways (64–66), which expression levels were significantly downregulated in carriers compared to non-carriers (Table 5). However, one kinase, CHUK/IKKα essential for non-canonical NFκB signaling pathway but dispensable for canonical NFκB signaling pathway (67) was expressed at a significantly higher level (3.1-fold) in carriers than in non-carriers, whereas the expression of two other kinases (IKKB and IKKG) crucial for the canonical pathway was downregulated by >3-fold at close to a significant level (FDR = 0.12 and 0.15), suggesting the differential expression was in favor of activation of non-canonical NFκB signaling pathway.

Interestingly, four signaling enhancers of the IRF3 and/or canonical NFκB pathways, OTUB1 (68), RNF128 (69), TGFB2-OT1 (70) and TRIM52 (71) were expressed at significantly lower levels in carriers than in non-carriers. By contrast, four inhibitors, HIVEP2 (72), NKIRAS1 (73), NLK (74) and NKRF (75) of the canonical NFKB signaling pathway were expressed 2.8 to 11.8-fold higher in carriers than in non-carriers. Additionally, two immune inhibitory microRNAs, MIR221 (77, 78) and MIR503HG (79) as well as one proinflammatory one, MIR155, which also has both anti-apoptotic activity (115, 116) and an inhibitory effect on interferon signaling (76), were upregulated in carriers. These findings suggest that the interferon, IRF3, MAPK and especially canonical NFκB signaling pathways could be negatively impacted by the differential gene expression during persistent FMDV infection.

### Immune Cell-Associated Genes

MFSD6 recognizes certain MHC-I molecules and mediates MHC-I restricted killing by macrophages (117). The expression levels of MFSD6and a non-classical MHC-I were 3.9 and 7.7 times lower in carriers compared to non-carriers (Table 6). Similarly, a ligand (NCR3LG1) of NK cell receptor 3 (NCR3) (80) was expressed at a significantly lower level in carriers than in non-carriers, whereas a NK cell cytotoxicity inhibitory soluble ligand (NID1) of NCR2 (81) was significantly upregulated by 12.1-fold in carriers vs. non-carriers. This differential expression could inhibit macrophage- and NK cell-mediated cytotoxicity in carriers.

Intraepithelial lymphocytes (IEL) are heterogenous T cells reside within the epithelial layer of mucosal and barrier tissues. CD69 is a receptor expressed by several subsets of tissue resident immune cells such as IEL. It plays a key role in regulating T cell differentiation and activities depending on the cells [86]. Three probes of CD69 consistently showed ~5-fold upregulated expression (FDR = 0.00) in carriers compared to non-carriers (Table 6). On the other hand, two genes (CD27 and CD244), playing important roles in activating T cells especially CD8+ cytotoxic T cells and NK cells (42, 47, 48), were expressed at significantly lower levels in carriers. Additionally, TNFRSF19 Inhibits TGFβ Signaling (84) and TGFβ signaling suppressed T cell cytotoxicity and promote immune tolerance (85), whose expression was significantly downregulated in carriers. The differential expression of these genes could inhibit IEL cytotoxicity.

T-bet is required for the development of CD8aa+ IEL (88, 89). T-bet expression increases in non-CD4+ T cell-helped resident memory T cells, which suppresses CD103 expression (87). The expression of T-bet was significantly upregulated in carriers compared to non-carriers, whereas CD103 expression was downregulated (*P* = 0.03). A probe of bovine immunoglobulin delta heavy chain constant region mRNA (NCBI accession #: AF411240) displayed significant 2.3-fold increased signal intensity in carriers than in non-carriers, indicating more of γδ T cells in the epithelium of carriers than in non-carrier. γδ T cells are known to have regulatory functions in mucosal immunity (86). These results indicate that T cells in the epithelium (also called as IEL), especially γδ T cells, might play a role in FMDV persistent infection.

RORC is a Th17-specific transcription factor. RORC and STAT3 promotes Th17 differentiation (90). TIAM1 forms a complex with RORC in the nuclear compartment of Th17 cells and together they bind and activate the IL17 promoter (91). TIAM1 expression was downregulated by 20.8-fold in carriers compared to non-carriers, whereas the expression levels of RORC and STAT3 were lower in carriers than in non-carriers (*P* ≤ 0.03). On the other hand, CD69, ETS1, IL33 and STAT5 are negative regulators of Th17 differentiation and activity (90, 92, 118, 119). The expression levels of these four genes were 4.4- to 14.7-fold higher in carriers than in non-carriers. Th17 cells increase the expression of IL33 receptor (IL1RL1) upon inflammation in mucosa and IL33 induces acquire immunosuppressive properties in Th17 cells (93). IL1RL1 expression was higher in carriers than in non-carriers (*P* = 0.02). These results suggest that the Th17 response could be suppressed in carriers.

Tr1 is an antigen-specific FOXP3^−^ regulatory T cell and CD49B and PRDM1 are Tr1 markers (96, 97). CD49B and PRDM1 expression was significantly higher in carriers than in non-carriers (Table 6). Two genes associated with Tr1 differentiation (EBI3 and MAF) (96, 120) were also expressed higher in carriers (*p* = 0.02 and 0.1, respectively). FOXP3 and ThPOK are a CD4+ T cell-specific and Treg-specific transcription factors, respectively. FOXP3+ Treg cells lose expression of ThPOK and FOXP3 after migration to the mucosal epithelium and convert to CD4+ intraepithelial lymphocytes (IEL) (88, 98). The expression levels of FOXP3 and ThPOK but not CD4 were significantly lower in carriers compared to non-carriers (Table 6), indicating increased recruitment of FOXP3+ Treg cells to the epithelium during the establishment of FMDV persistent infection.

### Apoptosis and Inflammatory Mediators

IPA pathway analysis indicated cell death and survival could be affected by the differential gene expression. Apoptosis is a well-known cell death mechanism that plays a role in immunity against viral infection. The expression of four pro-apoptotic genes (CASP3, BNIPL, BCL2L14, and BCL2L1) was significantly downregulated by 3.3- to 8.3-fold and one anti-apoptotic gene (BNIP2) upregulated by 9.2-fold in carriers compared to non-carriers (Table 7). CASP3 is a critical caspase in the down-stream of apoptosis pathways that is activated by eternal and external signals such as virus infection, death receptor ligands (TNF, TRAIL, etc.) and cell-mediated cytotoxicity. TP53 is a tumor suppressor gene and is the top upstream regulator detected in this study. Two TP53-interacting genes, TP53BP1 (TP53 binding protein 1) and TP53RK (TP53 regulating kinase), were expressed at significantly lower levels by > 7-fold in carriers than in non-carriers (Table 7). TRAF3 and TRAF6 downregulated in carriers (Table 5) could also inhibit viral RNA-induced apoptosis activated by RIG-I-like receptor-induced IRF3 mediated pathway (64). These findings suggest that apoptosis triggered by virus infection might be inhibited in the nasopharyngeal epithelium of FMDV carriers as detected with IPA analysis.

Four downregulated (PTGR1, PTGES, PTGES2, and PTGS1) and one upregulated (PLA2G2A) genes involved in leukotriene B4 (LTB4) and prostaglandin production (103–105) were expressed at higher levels in carriers than in non-carriers (Table 7). The differential expression suggests there is imbalance between LTB4 (↑) and prostaglandin (↓) production. Higher expression of LTB4R, Leukotriene B4 receptor 1 (5.7-fold at *P* = 0.02, Table 7), supports increased production of LTB4 in carriers. Interestingly, a probe of bovine immunoglobulin epsilon heavy chain constant region mRNA (NCBI accession #: AY221098) displayed 3.9-fold increased signal intensity in carriers than in non-carriers (P = 0.02), indicating higher levels of IgE in carriers and indirectly supporting the increased production of LTB4 based on the role of LTB4 in allergy (102). The LTB4 and prostaglandin imbalance has been reported to play a role in *Mycobacterium tuberculosis* persistent infection (121).

### Transcription Factor Binding Sites

TATA box sequences were detected in the proximal promoter regions of CXCL15, CXCL16 and CXCL17 but not in chemerin. There is a CpG island sequence overlapping transcription start sites of chemerin and CXCL16 genes. There was at least one transcription factor binding site (TFBS) for RELA (A transcription factor activated by the canonical NFκB signaling pathway) detected in the proximal promoters of all four of these chemokine genes (Table 7). There were at least two TFBS for IRF3 and three for STAT1::STAT2 in these proximal promoters except for the chemerin promoter. No TFBS for FOS::JUN, IRF3 or STAT1::STAT2 were found in the chemerin proximal promoter, but five NFKB1 (another transcription factor activated by the canonical NFκB signaling pathway) TFBS were detected in the region. Several TFBS for IRF3, NFκB, and STAT1::STAT2 overlap, suggesting that multiple transcription factors can bind to the same sequences to regulate the transcription at these sites. These predicted TFBS suggest that the transcription of these four chemokine genes could be regulated by the interferon, IRF3, MAPK and/or canonical NFκB signaling pathways.

## Discussion

Persistent infection is not unique to FMDV as most, if not all, picornaviruses can persistently infect cells both *in-vitro* and *in-vivo* (122). In some virus families, virus-specific factors promoting persistent infection have been described (e.g., in lymphocytic choriomeningitis virus Clone 13) (123), but it has been demonstrated that virus mutations or specific viral genomic characteristics are not the decisive factors in the establishment of persistent FMDV infection *in-vitro* or *in-vivo* (124, 125). To date, it is not clear which specific mechanisms allow FMDV to persist in some hosts and why available vaccines can protect animals from clinical disease while not preventing or curing subclinical (primary) or persistent infection of the upper respiratory tract. In the current study, we used Ingenuity Pathway Analysis (IPA) to analyze all DEG and found that immune cell trafficking could be significantly impacted by genes differentially expressed between the nasopharyngeal epithelium of FMDV carriers and non-carriers.

It is well-known that immune cell trafficking is controlled by chemokines, and these immune cells express various receptors to respond to chemokine gradients. In the mucosa, epithelial cells express several chemokines to recruit immune cells to control mucosal infections and maintain homeostasis (126). Among five chemokine genes that were downregulated in the nasopharyngeal mucosa of FMDV carriers, chemerin, CXCL15, CXCL16, and CXCL17 have been reported to be expressed by epithelial cells (32, 34, 127, 128). Our results (Figure 2) also show that the expression of these four genes was higher in the micro-dissected epithelium than in whole tissue macerates of samples from the corresponding anatomic sites.

The mucosal immune system is separated into inductive and effector sites based upon anatomical and functional properties (129). Antigen presenting cells (APC) such as dendritic cells bind antigens in effector sites and migrate to mucosa-associated lymphoid tissues (inductive sites) where they present antigen epitopes to T cells and induce a specific set of chemokine receptors on the T cells to specifically migrate back to the mucosa (the effector sites). Chemerin recruits plasmacytoid dendritic cells, immature myeloid dendritic cells, macrophages and natural killer cells by binding to three different receptors; ChemR23, GPR1, and CCRL2 (30, 130). Downregulated expression of chemerin in carriers could reduce recruitment of dendritic cells to the epithelium, which could hinder the induction and reactivation of an adapted immunity. A potentially reduced recruitment of dendritic cells in FMDV carriers was further supported by downregulated expression of four APC-expressed genes (CD276/B7-H3, CLEC9A/DNGR1, VISTA/B7-H5, and VTCN1/B7-H4 (Table 2).

CXCL16 and CXCL17 are ELR- CXCL chemokines. CXCL16 has a strong chemotactic activity for activated CD8+ T cells, CD4+ T cells, NKT cells and intraepithelial lymphocytes (IEL) but weak or no activity for unstimulated T cells (33, 35). The expression of CXCL16 receptor (CXCR6) on T cells in the lung is correlated with local protective immunity against *Mycobacterium tuberculosis* (131). Like CXCL16, CXCL17 is chemotactic for antigen-experienced memory CD8+ T cells such as memory T effector (Tem) and resident memory (Trm) cells (132). CXCR8 is the receptor of CXCL17. CXCL17 null mice developed fewer CXCR8+ CD8+ Tem and Trm cells and exhibited greater herpes virus replication and susceptibility to latent herpes infection in the mucosa compared to wild-type mice (132). Like chemerin, CXCL17 also recruits antigen presenting cells such as immature dendritic cells (36, 133). Significant downregulation of CXCL16 and CXCL17 could reduce recruitment of antigen-experienced T cells to the infected epithelium, which then reduced the killing of infected cells.

In contrast, the recruitment of NK cells into persistently infected epithelium might increase based on upregulated expression of CCL2, CD16, and two KLR receptors (Table 3). NK cells can kill FMDV infected cells and the cytotoxicity can be enhanced by cytokines such as IL-2, IL-15, IL-18, and IFN-α (134). IFNA, IFNL, and IL15 were expressed in significantly higher levels in carriers than in non-carriers; however, downregulated NCR3 ligand (NCR3LG1/B7-H6) and upregulated extracellular NCR2 ligand (NID1), a NK cell cytotoxicity inhibitor (81) could significantly reduce interaction between infected epithelial cells and NK cells and compromise the effectiveness of NK cells in killing of FMDV infected cells. Therefore, the killing of FMDV infected epithelial cells by both antigen-specific cytotoxic T cells and NK cells could be compromised as discussed earlier and later based on upregulated CD69.

Although killing of infected cells reduces virus replication, additional mechanisms such as extracellular traps and phagocytosis by neutrophils or macrophages are needed to limit virus spread and clear infection from the host. All ELR+ chemokines, including CXCL1, CXCL2, CXCL3, CXCL5, CXCL6, and CXCL8, have a chemotactic activity for neutrophils via binding to CXCR2 (27). CXCL6 and CXCL8 can also bind to CXCR1 (135). CXCR1 and CXCR2 are known to be predominately expressed on neutrophils (27, 40). Downregulated expression of ELR+ chemokines (Table 2), CXCR1 and CXCR2 and other neutrophil-expressed genes in carriers (Table 3) strongly supports reduced recruitment of neutrophils in carriers. Because neutrophils produce CCL23 (136), lower CCL23 expression in carriers also indirectly supports our hypothesis.

CXCL15 is another ELR+ CXCL chemokine reported in mice and has a strong chemotactic activity for neutrophils (32). CXCL15-null mice were more susceptible to *Klebsiella pneumoniae* infection than wild-type mice (137). We have recently identified a novel bovine CXCL15 in cattle (138). The expression of CXCL15 was significantly downregulated in carriers compared to non-carriers and total expression of all seven bovine ELR+ CXCLs was also 2.4-fold lower in carriers (Table 2). CXCL15 gene appears to be not functional in water buffalo (*Bubalus bubalis*) due to early stop codon mutation (138). Interestingly, there is limited evidence that Asian buffalo are more susceptible to persistent FMDV infection based on higher percentage of FMDV persistence in buffalo than in cattle in one study (139), which indirectly supports the role of CXCL15 in FMDV persistence.

It is well-known that neutrophils are the most numerous circulating immune cells and play a critical role in the first line of defense against infections by engulfing and destroying pathogens as well as secreting anti-microbials, cytokines and chemokines to recruit other immune cells (140, 141). Neutrophils can also release neutrophil extracellular traps (NET) to immobilize and inactivate viruses (142). NET are extracellular fibril matrices composed of granule proteins and chromatin released by activated neutrophils (143), which has been reported to play a role in eliminating virus infections (144, 145).

To understand why the expression of chemerin, CXCL15, CXCL16, and CXCL17 chemokines was downregulated in carriers, we first examined the expression of cytokines. Unexpectedly, all significantly differentially expressed cytokine genes were upregulated in carriers and all are proinflammatory (Table 4). Upregulated expression of the cytokines probably was due to increased recruitment of monocytes into the epithelium of FMDV carriers based on upregulated CCL2, C5AR1, CD16, CD300, CD300LD, MIR155HG, and MMD expression (Tables 2, 3, 5). MMD overexpression has been shown to increase TNF production in a macrophage cell line (62). Co-culture of IL-15-stimulated NK cells with blood mononuclear cells induced TNF production in macrophages, which in turn induced CD69 expression on lymphocytes (146). CD69 and IL15 were also significantly upregulated in carriers in the current study (Table 4).

TNF was the most upregulated cytokine in carriers. The expression of another TNF cytokine (TNFSF10 or TRAIL) also known to be able to kill virus-infected cells via death receptor signaling to induce apoptosis (147) was also upregulated in carriers. The expression levels of TNF and TRAIL were higher in the lung (the tissue susceptible to acute FMDV infection but resistant to persistent infection) than in pharyngeal tissues (20). It may be concluded that TNF and TRAIL probably are not very effective in killing FMDV infected cells in carriers because apoptosis induced by these two cytokines could be suppressed by the significantly down-regulated (6.9-fold) expression of a key apoptosis activator, CASP3 (99) and other apoptosis-related genes listed in Table 7. Interestingly, TP53 (a tumor suppresser gene) was the top regulator detected in this study. TP53BP1 interacts with TP53 and NFκB and can sensitize breast cancer cells to apoptosis induced by TNF treatment (100). TP53RK is a TP53 kinase that can phosphorylate and activate TP53 (101). Therefore, significantly downregulated CASP3, TB53RK and TP53BP1 together with TRAF3 and TRAF6, the signal transducers in RIG-I-induced IRF3 mediated pathway of apoptosis (64), strongly support the suppression of apoptosis induced by death receptor signaling, virus RNA and cell-mediated cytotoxicity in carriers.

IL33 was the second most upregulated (14.7-fold) cytokine in FMDV carriers. The receptor of IL33 is constitutively expressed on mast cells, group 2 innate lymphoid cells and Tregs. IL33 plays a key regulatory role in mucosal immunity (95). This cytokine promotes the accumulation and function of myeloid-derived suppressor cells (148) and regulatory T-cells in the intestine (149), induces alternative activation of macrophages (150) and has a potent suppressive effect on innate antiviral immunity (151). Epithelial cells are the major source of IL33 production in the intestine during inflammation (93). Stimulation of IL33 changed the Th17 expression profile in favor of an immunosuppressive phenotype (93). The expression of IL33 in macrophages can be induced by aryl hydrocarbon receptor (AHR) (152). It is well-known that AHR plays a critical role in mucosal immunity (153).

Interestingly, CD69 can increase uptake of L-tryptophan through LAT1-CD98 for converting tryptophan into AHR ligands to activate AHR signaling (83) and CD69 was one of the most consistently upregulated genes in carriers (Table 6). Significantly downregulated CD98/LAT1 in carriers (Table 6) could reduce the effect of upregulated CD69 on AHR ligand production. However, CD69 also has a broad immune suppressive effect via receptor signaling (82). The expression of CD69 and miR-155 (also upregulated in carriers in this study) are coregulated in a positive-feedback loop to promote Treg cell differentiation (154). Increased CD69 expression enhances immunosuppressive function of regulatory T-cells (155, 156), suppressed Th17 cell differentiation (82, 118, 157) and T cell cytotoxicity (158).

The expression of CD69 on lymphocytes in the gut appears to depend on the microflora because germ-free mice and the ablation of microflora decreased CD69 expression (159, 160). Sustained expression of CD69 on activated T lymphocytes depends on non-canonical NFκB signaling (161). Interestingly, our results indicate that the activation of upregulated IL1s and TNFs in NFκB signaling pathways were routed to the non-canonical pathway due to significantly upregulated IKKα and downregulated IKKβ and IKKγ (Table 5). The activation of the non-canonical pathway is known to play a role in peripheral immune tolerance and secondary lymphoid tissue development (71). FMDV persistent infection was observed in the follicle-associated epithelia of the nasopharyngeal mucosa (7, 10).

Next we examined the differential expression of genes involved in cytokine signaling pathways. The results listed in Table 5 could significantly suppress the expression of downregulated chemokines in carriers. IL17A and IL17F activate NFκB signaling pathways and play an important role in mucosal immunity via inducing chemokine expression to recruit neutrophils (162, 163). It induces neutrophil recruiting ELR+ CXCL chemokines in epithelial cells (164) and increases the stability of CXCL1 and CXCL5 mRNA (165, 166). Interestingly, the expression of IL17RA (the IL17A and IL17F receptor) was significantly downregulated in carriers, which could inhibit the response of epithelial cells to IL17 stimulation. Additionally, downregulated NFkB signaling enhancers and CHUK/IKKα and upregulated signaling inhibiters, IKKβ and IKKγ (Table 5) could play a significant role in the downregulated chemokine expression.

Third, we examined more DEG that could suppress the immune response. DEG listed in Table 6 indicate that the cytotoxicity of macrophages, NK cells and CD8+ cytotoxic T cells and especially the Th17 response could be suppressed in carriers. As stated earlier for IL33 and CD69, there were two more DEG upregulated in carriers (ETS1 and STAT5B) known to suppress Th17 differentiation (90, 92, 119), whereas TIAM1 needed for IL17 expression (91) was downregulated by 20-fold in carriers (Table 6). These results could explain why upregulated Th17-stimulatory cytokines did not result in higher IL17 expression in carriers (Table 4). Therefore, the suppression on the Th17 response could also play a role in FMDV persistent infection.

Finally, we predicted the TFBS in the proximal promoters of the four epithelium-expressed chemokines; chemerin CXCL15, CXCL16, and CXCL17, to infer if inhibition of the signaling pathways could reduce the expression of these four chemokine genes. The results of the promoter analysis indicate that the expression of all four chemokines relies on the activation of canonical NFκB (NFKB1 and RELA) transcription factors, whereas CXCL15, CXCL16, and CXCL17 may also depend on IRF3, interferon and MAPK signaling pathways. CXCL15 expression has been shown to increase under inflammatory conditions such as antigen and LPS stimulation and infection (32, 167). IFNγ and TNF stimulates CXCL16 and CXCL17 RNA expression in cultured cells (126, 168). IFNγ and TNF appear to have a synergetic effect on CXCL16 expression, indicating that multiple transcription factors act together to regulate expression of this chemokine. Interestingly, the differential expression of DEG listed in Table 5 could inhibit these signaling pathways especially canonical NFκB signaling pathway, which is known to be activated by IL-17, IL-33, and TNF as stated earlier.

Our previous study using whole pharyngeal tissues showed cytokine and Tr1-expressed genes being differentially expressed between carriers and non-carriers (21). In current study, only two Tr1 marker genes (CD49B and PRDM1) were significantly upregulated in carriers, and one transcription factor (MAF) and one cytokine (IL27B) critical for Tr1 differentiation (96) were upregulated (p ≤ 0.02) at non-significant levels. The expression of IL10, a typical cytokine produced by Tr1 cells, was not differentially expressed. EBI3, one of an immunosuppressive cytokine IL35 dimer, was upregulated (*p* = 0.02) at a non-significant level. The differences could be due different tissue sampling in the study. Most regulatory T cells reside in the lamina propria instead of the epithelia (129).

A higher significant threshold (FDR ≤ 0.05) was used in the previous study of this set of microarray data used in current study (22). Most of the DEG listed in current study was not evaluated in the previous study. Immunohistochemistry analysis using anti-CD3D, anti-CD8A and anti-γδTCR antibodies showed no differences in the numbers of the detected cell populations between carriers and non-carriers (22). These antibodies could not distinguish between naïve and antigen-experienced effector T cells. The results generally agree with the results found in current study of no differences in overall T cell population sizes, though the results of the current study suggest increased naïve T cell/natural IEL- and decreased effector T cell-recruitment.

**Figure 3:**
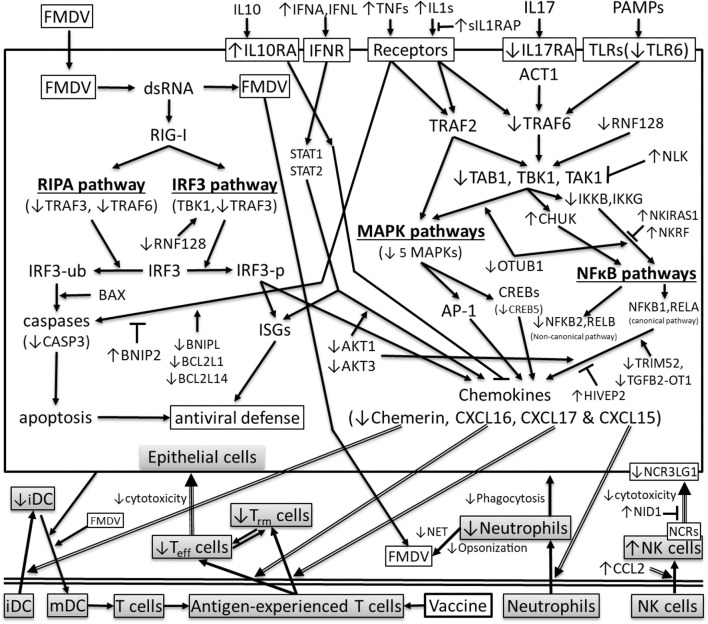
Differentially expressed genes in cytokine signaling pathways that could affect the transcriptions of chemerin, CXCL15, CXCL16, and CXCL17 in the pharyngeal epithelial cells of FMDV carriers (↑ or ↓: significantly up- or downregulated in carriers compared to non-carriers, respectively; arrow: stimulation;⊤ inhibition; iDC and mDC: immature and mature DC; IRF3-ub and IRF3-p: ubiquitinated and phosphorylated IRF3, respectively; ISGs: interferon-stimulated genes; MAPKs: mitogen-activated protein kinases; NCRs: NK cell receptors; NET: neutrophil extracellular traps; RIPA: RIG-I-like receptor-induced IRF3 mediated pathway of apoptosis; T_eff_: effector T cells; T_rm_: memory T cells; double line arrow: cell recruitment; double line: separation between epithelium and other tissues).

In conclusion, the IPA pathway analysis suggests that the detected differential gene expression could affect cell death and survival, immune cell trafficking and hematological system development and function. Four chemokines (chemerin, CXCL15, CXCL16, and CXCL17) recruiting neutrophils, antigen-experienced T cells and/or dendritic cells were downregulated in FMDV carriers, whereas macrophage and NK cell recruiting CCL2 was upregulated. Other DEG support the differential expression of the chemokines, as shown throughout these analyses. Although all differentially expressed cytokines were upregulated and proinflammatory, the DEG in signaling pathways suggested that the interferon, IRF3, MAPK, and NFκB signaling pathways especially the canonical NFκB pathway could be inhibited in carriers. The TFBS predicted from the proximal promoters indicated that the expression of these downregulated chemokines depends on the activation of these signaling pathways. DEG such as CASP3, CD69, IL17RA, IL33, NCR3LG1, NID1, TP53BP1, TRAF3, and TRAF6 indicated that the Th17 response, NK cell cytotoxicity and apoptosis could be suppressed in carriers. Therefore, based on our results and published gene functions, we hypothesize that (1) under-expression of chemokines that recruit neutrophils, antigen-experienced T cells and dendritic cells, (2) blocking NK cell binding to infected cells and (3) suppression of cell-mediated cytotoxicity-, death receptor signaling- and viral RNA-induced apoptosis compromised virus clearance and allowed FMDV to persist as shown in Figure 3. These hypothesized mechanisms indicate that vaccines may not be effective in curing FMDV persistent infection. This study provides novel insights for further investigation.

## Data Availability Statement

The datasets generated for this study can be found in the Michael Eschbaumer, http://www.ncbi.nlm.nih.gov/geo/query/acc.cgi?acc=GSE104058.

## Ethics Statement

The animal study was reviewed and approved by Plum Island Institutional Animal Care and Use Committee (Protocol numbers 209-12-R, 209-15-R).

## Author Contributions

JZ designed the bovine microarray and the microarray experiment, conducted gene annotation, bioinformatic analysis, and wrote the manuscript. JA and CS conceived and executed the primary animal studies and laboratory analysis. CS collected and analyzed the animal samples and performed tissue microdissection. EB performed gene annotation. ME performed microarray analyses and statistical analysis of the data. All authors reviewed and edited in the manuscript.

## Conflict of Interest

The authors declare that the research was conducted in the absence of any commercial or financial relationships that could be construed as a potential conflict of interest.
